# Long-Term Trajectories of Cognitive Disability Among Older Adults Following a Major Disaster

**DOI:** 10.1001/jamanetworkopen.2024.48277

**Published:** 2024-12-02

**Authors:** Huaqin Hu, Buqun Li, Hiroyuki Hikichi, Ichiro Kawachi, Xiaoyu Li

**Affiliations:** 1 Department of Sociology, Tsinghua University, Beijing, China; 2 Division of Public Health, Kitasato University School of Medicine, Kanagawa, Japan; 3 Department of Social and Behavioral Sciences, Harvard T. H. School of Public Health, Boston, Massachusetts

## Abstract

**Importance:**

Older adults are at high risk for cognitive impairments following natural disasters, but the persistence of such effects is not well understood.

**Objective:**

To track older adults’ postdisaster cognitive disability trajectories over a decade and examine associations of cognitive disability with disaster damage exposure.

**Design, Setting, and Participants:**

This prospective cohort study used predisaster baseline surveys conducted in August 2010 and 4 follow-ups approximately every 3 years until November 10, 2022. Invitations were mailed at baseline to all citizens 65 years or older in Iwanuma City, Japan, a coastal municipality significantly impacted by the 2011 Great East Japan Earthquake and Tsunami. A total of 1988 respondents who were cognitively independent (ie, without cognitive disability) before the disaster completed the 4 postdisaster cognitive assessments.

**Exposures:**

Experiences of residential damage, worsening financial conditions, loss of loved ones, and disruption in health care services. A composite score quantified these experiences, with higher scores indicating greater overall damage.

**Main Outcomes and Measures:**

Cognitive disability level was assessed using a standardized in-home evaluation under a national long-term care insurance scheme. Trained investigators evaluated participants’ activities of daily living, cognitive function, and presence of mental and/or behavioral disorders. Latent class growth analyses (LCGA) identified distinct cognitive disability trajectories. Primary outcomes consisted of inclusion in these trajectories.

**Results:**

The analytic sample consisted of 1988 participants who were cognitively independent at baseline, with a mean (SD) age of 72.4 (5.4) years, of whom 1159 (58.3%) were female. LCGA identified 3 cognitive disability trajectories: high and gradual deterioration (277 [13.9%]; high levels of cognitive disability with increasing impairment over time), low and progressive deterioration (541 [27.2%]; low levels of cognitive disability with accelerated decline in cognitive function over time), and low and stable (1170 [58.9%]; low levels of cognitive disability that remained stable). In multinomial regression analyses with the low and stable trajectory serving as the reference, housing damage (adjusted odds ratio [AOR], 2.52; 95% CI, 1.26-5.04), worsening financial conditions (AOR, 1.83; 95% CI, 1.15-2.90), and disruption in health care services (AOR, 1.76; 95% CI, 1.03-2.99) were associated with high and gradual deterioration. Worsening financial conditions (AOR, 1.38; 95% CI, 1.01-1.90) and higher composite damage scores (AOR, 1.16; 95% CI, 1.01-1.34) were associated with low and progressive deterioration. These associations no longer remained after adjusting for postdisaster depressive symptoms.

**Conclusions and Relevance:**

In this cohort study of older adults who survived the 2011 Great East Japan Earthquake and Tsunami, disaster-related exposures were associated with worse long-term cognitive disability trajectories. These findings could inform tailored interventions to preserve cognitive function in older disaster survivors.

## Key Points

**Question:**

Is exposure to a major natural disaster associated with long-term trajectory of cognitive disability among older adults?

**Findings:**

In this cohort study of 1988 older adults with cognitive assessments over a decade, experiences of housing damage, worsening financial conditions, disruption in health care services, and greater extent of overall damage were associated with 2 cognitive deterioration trajectories. These associations did not remain when adjusting for postdisaster depressive symptoms.

**Meaning:**

These findings suggest that disaster-related damage may impact the long-term cognitive trajectory among older adults, which could guide tailored interventions to preserve cognitive function in this population.

## Teaser

This cohort study identifies longitudinal trajectories of cognitive disability among older adults using data from the Iwanuma Study after the 2011 Great East Japan Earthquake and Tsunami and examines how various types of disaster damage are associated with these trajectories.

## Introduction

Natural disasters are unpredictable and devastating events, causing severe consequences for human lives, health, and well-being. According to the National Geophysical Data Center, the world experienced peak disaster death tolls in 2004 and 2010, with 228 792 and 319 319 recorded deaths, respectively; earthquake-related deaths, including those resulting from tsunamis triggered by earthquakes, accounted for 93% and 69%, respectively, of all disaster-related fatalities in those years.^1^ Moreover, these disasters can lead to long-term physical and mental health problems, such as functional disability and posttraumatic stress disorder.^2,3^ One often overlooked consequence of experiencing disasters is cognitive decline observed among older adults, thereby increasing dependency and affecting quality of life.^4^

Previous studies have documented a high prevalence of dementia, or accelerated cognitive decline, among older adults who experience natural disasters.^5,6^ Some plausible mechanisms have been discussed, such as the direct effects of psychological trauma and residential dislocation leading to social isolation.^7^ However, the long-term impact of disaster damage on cognitive function in older adults remains poorly understood. Furthermore, most prior analyses either did not control for predisaster conditions or used retrospectively collected predisaster information that might be limited by recall bias,^8,9^ which may affect the validity of the inferences.

Previous studies^10,11^ have revealed that distinct subgroups may experience different cognitive trajectories over time across both community samples and clinical cohorts. However, little is known about whether there are latent classes of disaster survivors whose cognitive functions follow unique longitudinal patterns. Therefore, the present study identified longitudinal trajectories of cognitive disability among older adults using data from the Iwanuma Study after the 2011 Great East Japan Earthquake and Tsunami. We examined how various types of disaster damage were associated with these trajectories, which could aid in the development of effective postdisaster interventions.

## Methods

### Participants

Our cohort study used data from the Iwanuma Study, which is part of the Japanese Gerontological Evaluation Study (JAGES). Detailed information about JAGES has been described in a previous study.^12^ Briefly, JAGES is a nationwide cohort study initiated in 2010 to identify factors associated with healthy aging among older adults, comprising 169 215 community-dwelling individuals across 31 municipalities, with Iwanuma City as one of the field sites.

In August 2010, all community-dwelling older adults 65 years or older residing in Iwanuma City (N = 8576) were invited to participate in the baseline survey. A total of 5058 individuals responded (response rate: 59.0%), providing information regarding their personal characteristics, socioeconomic status, mental health, sleep health, and other relevant factors.^13^ Seven months after the baseline survey, the city was struck by a catastrophic earthquake and tsunami. Follow-up surveys were conducted in 2013, 2016, 2019, and 2022 (eFigure 1 in Supplement 1). The survey protocol was reviewed and approved by the human subjects committees of Harvard T. H. Chan School of Public Health, Boston, Massachusetts; Tohoku University, Sendai, Japan; Nihon Fukushi University, Mihama, Japan; and Chiba University, Chiba, Japan. This study follows the Strengthening the Reporting of Observational Studies in Epidemiology (STROBE) reporting guideline. Written informed consent was obtained at the time of survey collection.

**Figure 1.**
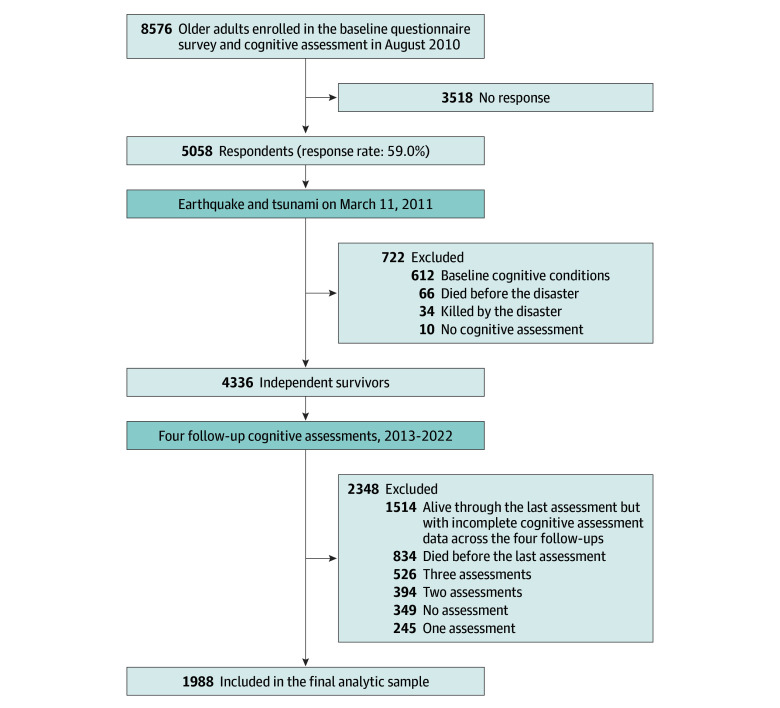
Flowchart of the Analytic Sample

In-home cognitive disability assessments from the Japanese long-term care insurance (LTCI) scheme were linked to the individual participants in the survey. After the baseline survey in 2010, a total of 110 participants moved from Iwanuma City (eTable 1 in Supplement 1). Following the disaster in 2011, 4336 participants who were cognitively independent (ie, no cognitive disability) at baseline survived. Of these, 1988 adults with complete cognitive assessment data from all 4 follow-ups were included in the main analyses. The 11-year follow-up rate of 45.8% was comparable to those of other cohort studies of community-dwelling older adults.^14^ A detailed flowchart of the analytic sample is presented in Figure 1.

### Measures

Our outcome of interest is the level of cognitive disability after the disaster measured by a standardized in-home assessment under the LTCI scheme.^15^ Registration in the LTCI is mandatory in Japan. Long-term care applicants undergo eligibility assessments by trained investigators from each municipality. The assessment includes evaluations of activities of daily living and instrumental activities of daily living, cognitive function (including short-term memory, orientation, and communication), as well as the presence of mental and behavioral disorders (eg, delusions of persecution, confabulation, and soliloquy). Following this comprehensive evaluation, individuals are classified into 8 levels (0 indicates independent; 7, requires specialized medical treatment in a long-term care facility) according to cognitive disability severity (eTable 2 in Supplement 1). Respondents who did not require care services in each follow-up period (ie, who were assessed to be cognitively independent) were assigned a score of 0. The assessment of cognitive disability levels is strongly correlated with the Mini-Mental State Examination (Spearman rank correlation ρ = −0.73; *P* < .001) and the Clinical Dementia Rating scale (specificity and sensitivity, 0.88; *P* < .001).^16^ For the trajectory analyses, we normalized the cognitive disability levels using a transformation process similar to that applied in cognitive studies with the Mini-Mental State Examination and mapped the resulting scores onto a scale ranging from 0 to 100.^17^ Specifically, a latent process mixed model was used to generate normalized scores, preserving the original direction of the assessments, where higher values indicate greater cognitive disability. The transformation adjusted the distances between consecutive levels to correct for curvilinearity, and the normalized scores were then mapped onto a scale ranging from 0 to 100 for ease of interpretation.

The first postdisaster follow-up survey (conducted in 2013) assessed disaster damage exposures in Iwanuma City, including housing damage, worsening financial conditions, loss of loved ones, and disruption in health care services. Property inspectors classified housing damage into 5 levels: no damage and partially, half, mostly, and completely destroyed. We derived a binary variable for housing damage (1 indicates mostly or completely destroyed; 0, no damage or less severe damage) based on prior evidence.^18^ Participants’ reports of postdisaster changes in financial conditions were used to establish a binary variable for worsening financial conditions (1 indicates worse or much worse; 0, not changed, improved, or improved a lot). Loss of loved one(s) assessed whether the respondent lost relatives, friends, or pets due to the disaster (1 indicates yes; 0, no). Disruption in health care services was defined as “unable to receive a medical examination because of the earthquake” (1 indicates yes; 0, no). A composite disaster damage score was derived as the sum of individual scores for each type of disaster damage experienced (range, 0-4).

The analyses controlled for demographic variables measured at baseline as potential confounders, including age, sex, equivalized income, educational level, marital status, and employment status. Household income was equalized by dividing the annual gross income by the square root of the number of household members. The analyses also controlled for 2 postdisaster variables assessed in the first follow-up survey: depressive symptoms measured by the Geriatric Depression Scale–15^19^ and a composite sleep score calculated according to the multidimensional sleep health framework^20^ (eTable 3 in Supplement 1).

### Statistical Analysis

Descriptive statistics were used to describe the study sample. Discrete cognitive disability trajectories were identified through unconditional latent class growth analysis (LCGA), a method that discerns latent classes characterized by similar developmental patterns of the outcome of interest over time.^21^ The elapsed time was measured in years since disaster. We followed a 2-step process to complete the LCGA.^22^ First, we hypothesized that cognitive disability would follow 2 to 5 distinct and clinically meaningful trajectories. Thus, we fit 4 models, each specifying a different number of trajectories (range, 2-5). We selected the best-performing model using criteria including log likelihood, Akaike information criterion, Bayes information criterion, and clinical relevance and assessed the model adequacy with mean of posterior probabilities and entropy.^23^ Next, we fixed the number of trajectories (determined in the prior step) and ran models that differed in polynomial form of each trajectory group (eg, linear and quadratic), identifying the model with the optimal combination of trajectory group functional forms using Bayes information criterion values. Model selection process was presented in eTables 4 and 5 in Supplement 1.

We then examined differences in baseline characteristics and disaster damage exposures across latent class trajectory groups. Fisher exact test was used for categorical variables and independent *t* test for continuous variables. Multinomial logistic regression analyses were used to examine the associations between disaster damage exposures and cognitive disability trajectory, adjusting for baseline covariates. Two postdisaster variables, depressive symptoms and sleep measured at the first follow-up survey, were then added into the regression models.

To assess the robustness of our findings, we performed sensitivity analyses by rerunning the trajectory and regression analyses on a larger sample, including participants with at least 1 cognitive assessment during the 4 follow-ups. All analyses were performed using statistical software R, version 4.1.3 (R Program for Statistical Computing). The lcmm package was used for the LCGA. Two-sided *P* < .05 indicated statistical significance.

## Results

The analytic sample consisted of 1988 participants who were cognitively independent at baseline, with a mean (SD) age of 72.4 (5.4) years; 1159 (58.3%) were female and 829 (41.7%) were male. The Table summarizes the characteristics and disaster damage exposures. Among those with information available, about half of the participants had 10 to 12 years of education (907 of 1932 [46.9%]), and most were married (1456 of 1926 [75.6%]). The mean (SD) equivalized income of participants was ¥2 360 000 (¥1 390 000 [¥152 439 equals US $1]), and most participants were not working (1419 of 1774 [80.0%]). Many lost loved ones (809 of 1958 [41.3%]) due to the disaster, while fewer reported worsening financial conditions (444 of 1924 [23.1%]), disruption in health care services (237 of 1958 [12.1%]), and housing damage (148 of 1919 [7.7%]). The mean (SD) composite damage score was 0.8 (0.9). Compared with the excluded sample, the analytic sample was younger, was more likely to be married, and had better baseline health status. In the excluded sample, participants who died before the last assessment showed the worst baseline cognitive disability levels compared with those alive through the last assessment, including those who moved and did not move from the area (eTable 1 in Supplement 1).

**Table.** Baseline Characteristics and Disaster Damage Exposures of the Analytic Sample

Characteristic	Cognitive trajectory, No. (%)^a^			
	All (N = 1988)	Low and stable (n = 1170)	Low and progressive deterioration (n = 541)	High and gradual deterioration (n = 277)
Age, mean (SD), y	72.4 (5.4)	70.1 (4.2)	74.5 (5.0)	77.7 (5.2)
Sex				
Female	1159 (58.3)	608 (52.0)	331 (61.2)	220 (79.4)
Male	829 (41.7)	562 (48.0)	210 (38.8)	57 (20.6)
Educational level, y				
≤9	602 (31.2)	301 (26.1)	183 (35.1)	118 (45.7)
10-12	907 (46.9)	570 (49.4)	234 (44.9)	103 (39.9)
≥13	423 (21.9)	282 (24.5)	104 (20.0)	37 (14.3)
Marriage status				
Married	1456 (75.6)	932 (81.2)	377 (72.4)	147 (57.2)
Unmarried	470 (24.4)	216 (18.8)	144 (27.6)	110 (42.8)
Equivalized annual income, ¥10 000, mean (SD)^b^	236 (139)	243 (138)	228 (136)	214 (147)
Employment status				
Working	355 (20.0)	276 (25.4)	61 (13.1)	18 (8.1)
Not working	1419 (80.0)	809 (74.6)	405 (86.9)	205 (91.9)
Loss of loved one(s)	809 (41.3)	494 (42.2)	227 (42.0)	88 (35.5)
Housing damage	148 (7.7)	74 (6.4)	44 (8.4)	30 (12.4)
Worsening financial conditions	444 (23.1)	248 (21.4)	135 (25.5)	61 (25.8)
Disruption in health care services	237 (12.1)	119 (10.2)	70 (13.0)	48 (19.4)
Composite damage score, mean (SD)^c^	0.8 (0.9)	0.8 (0.9)	0.9 (1.0)	0.9 (1.0)

^a^ All participants were 65 years or older. Unless otherwise indicated, data are expressed as number/percentage (%) of participants. Percentages have been rounded and may not sum to 100. Owing to missing data, numbers may not sum to column totals. High and gradual deterioration indicates high levels of cognitive disability with increasing impairment over time; low and progressive deterioration, low levels of cognitive disability with accelerated decline in cognitive function over time; and low and stable, low levels of cognitive disability that remained stable.

^b^ ¥152 439 Equals US $1.

^c^ Derived as the sum of individual scores for each type of disaster damage experienced (range, 0-4), with higher scores indicating worse experience of disaster damage.

**Figure 2.**
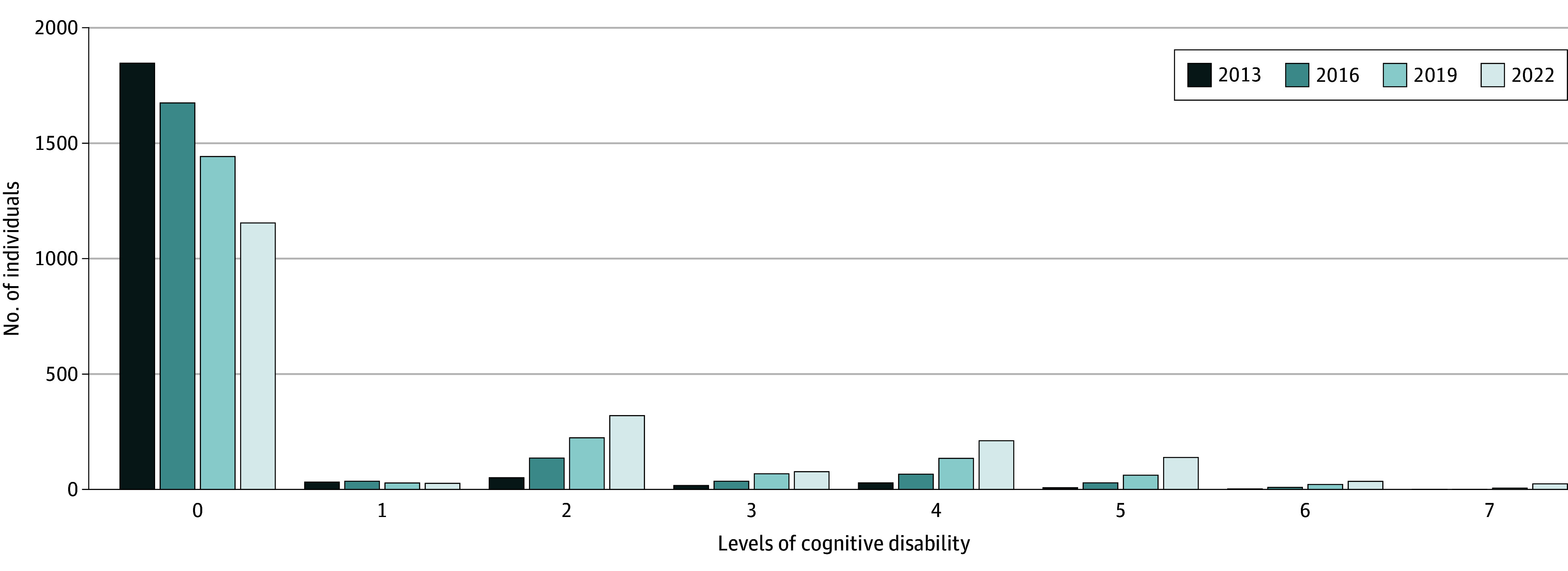
Distribution of Cognitive Disability Levels Across the Follow-Up Period (2013-2022) Comprehensive evaluation findings are classified into 8 levels ranging from 0 (independent) to 7 (requires specialized medical treatment in a long-term care facility) according to cognitive disability severity.

Figure 2 showed a right-skewed distribution of cognitive disability levels among participants from 2013 to 2022. While most of the participants maintained cognitive independence (level 0) each year, the number of participants with cognitive disabilities (levels 1-7) gradually increased over time. In addition, 525 participants (26.4%) showed cognitive disability levels above level 1 across two consecutive assessments.

**Figure 3.**
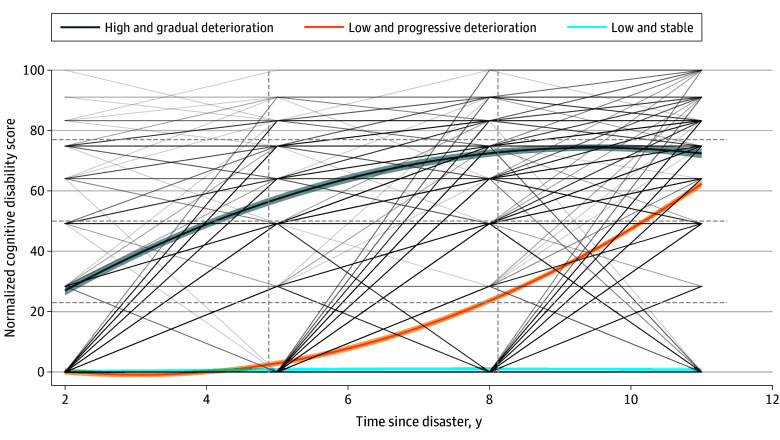
Trajectories of Normalized Cognitive Disability Score Identified by Latent Class Growth Analysis Of 1988 participants included in the analysis, 277 (13.9%) experienced the high and gradual deterioration pattern (high levels of cognitive disability with increasing impairment over time); 541 (27.2%), the low and progressive deterioration pattern (low levels of cognitive disability with accelerated decline in cognitive function over time); and 1170 (58.9%), the low and stable pattern (low levels of cognitive disability that remained stable; reference). The narrow gray lines represent individual trajectories of cognitive disability, showing variability across individuals. The shaded areas around the main trajectory lines indicate 95% CIs, capturing the range of uncertainty around each trajectory.

Three distinct developmental patterns of cognitive disability over a decade following the disaster were identified among the older adult survivors (Figure 3). The first pattern, labeled high and gradual deterioration, included individuals who initially presented with high levels of cognitive disability and experienced increasing impairment over time (277 [13.9%]). The second pattern, labeled low and progressive deterioration, comprised individuals who began with low levels of cognitive disability but underwent accelerated decline in cognitive function over time (541 [27.2%]). The low and stable pattern started with low levels of cognitive disability and remained stable throughout the observation period (1170 [58.9%]).

Multiple differences in baseline characteristics and disaster damage exposures were observed across cognitive disability trajectory groups (Table). The group with high and gradual deterioration had the oldest mean (SD) age (77.7 [5.2] years), the highest percentage of women (220 of 277 [79.4%]), the lowest educational level (37 of 258 [14.3%] with ≥13 years of education), the fewest married individuals (147 of 257 [57.2%]), and the lowest mean (SD) income (¥2 140 000 [¥1 470 000]). Conversely, both groups with low and stable and low and progressive deterioration patterns presented as more socioeconomically advantaged.

**Figure 4.**
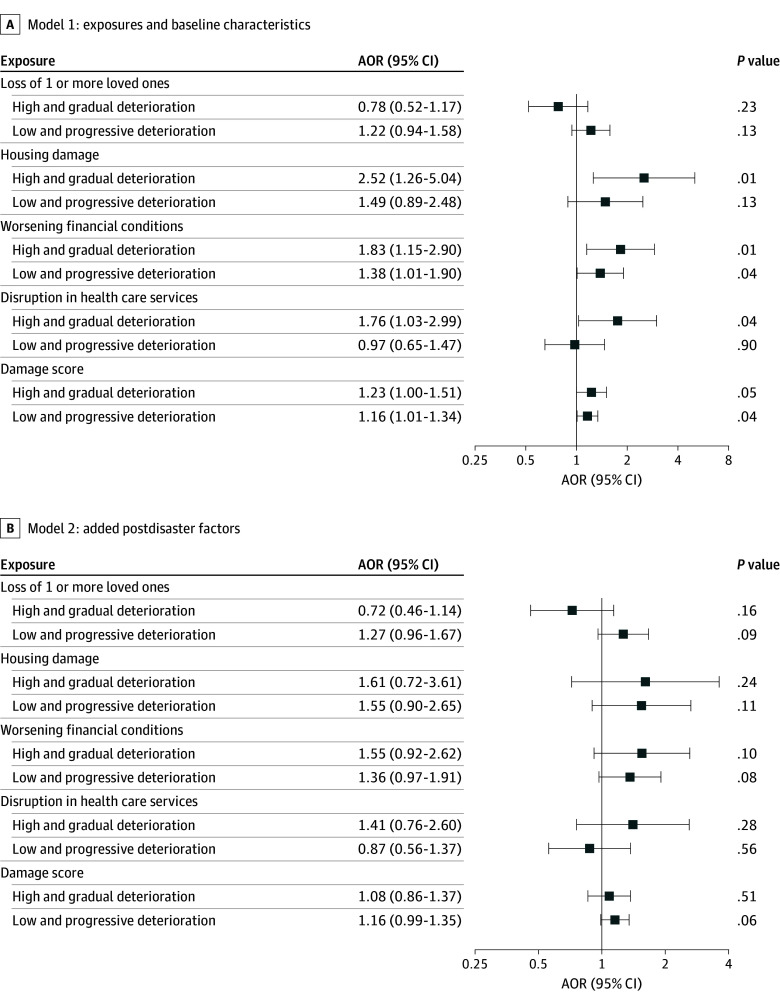
Associations Between Disaster Damage Exposures and Cognitive Trajectory Membership The low and stable cognitive pattern (low levels of cognitive disability that remained stable) constituted the reference group. Model 1 included disaster damage exposures and baseline characteristics; model 2 included disaster damage exposures, baseline characteristics, and postdisaster depressive symptom score and composite sleep score in 2013. High and gradual deterioration indicates high levels of cognitive disability with increasing impairment over time; low and progressive deterioration, low levels of cognitive disability with accelerated decline in cognitive function over time. AOR indicates adjusted odds ratio.

The adjusted associations between disaster damage exposures and cognition trajectories (low and stable constituted the reference group) from multinomial regression models are shown in Figure 4. Housing damage (adjusted odds ratio [AOR], 2.52; 95% CI, 1.26-5.04), worsening financial conditions (AOR, 1.83; 95% CI, 1.15-2.90), and disruption in health care services (AOR, 1.76; 95% CI, 1.03-2.99) were associated with increased odds of being in the high and gradual deterioration group relative to the low and stable group. Facing worsening financial conditions (AOR, 1.38; 95% CI, 1.01-1.90) was positively associated with the low and progressive deterioration pattern relative to the low and stable pattern. Additionally, as the composite damage score rose, there was a corresponding increase in the odds of individuals being classified into the low and progressive deterioration group (AOR, 1.16; 95% CI, 1.01-1.34) compared with the low and stable group.

After the introduction of postdisaster factors (depressive symptoms and sleep) into regression models, previous associations between disaster damage exposures, including the composite damage score, and cognitive disability trajectories, no longer remained (Figure 4). However, a 1-point increase in depressive symptom scores increased the odds of membership in the low and progressive deterioration group to 1.21 (95% CI, 1.13-1.29) and in the high and gradual deterioration group to 1.05 (95% CI, 1.00-1.10) (eTable 6 in Supplement 1). However, composite sleep score did not show associations.

In the sensitivity analyses, we reran both the trajectory and regression analyses on a larger sample of participants, including those with at least 1 cognitive assessment across the 4 follow-ups (n = 3854). The same 3 trajectory patterns were identified, with participant distributions across these trajectories closely matching those from the complete dataset (eFigure 2 in Supplement 1). The regression results remained largely unchanged, although some estimates became statistically insignificant. The composite damage score, for example, was not associated with increased odds of being in the low and progressive deterioration group (AOR, 1.06; 95% CI, 0.94-1.18) relative to the low and stable group (eTable 7 in Supplement 1).

## Discussion

This cohort study tracked cognitive disability in older adult survivors of the 2011 Great East Japan Earthquake and Tsunami and has, to our knowledge, one of the longest follow-up periods of cognitive disability after the disaster. These individuals were all cognitively independent before the disaster. Three distinct cognitive disability trajectories were revealed: low and stable (58.9%), low and progressive deterioration (27.2%), and high and gradual deterioration (13.9%). Disaster damage exposures such as housing damage, worsening financial conditions, disruption in health care services, and a higher composite damage score reflecting greater overall damage were associated with increased odds of belonging to the groups who experienced deterioration. Postdisaster depressive symptoms eliminated the associations between disaster exposures and cognitive disability trajectories. These findings shed light on not just the persistent cognitive repercussions of natural disasters, but also the nuanced differences between subgroups.

To contextualize these findings within a broader framework, it is informative to compare the cognitive disability outcomes of our cohort with those observed in community-dwelling older adults. Mimmack et al^24^ defined clinical progression as the first instance where 2 consecutive evaluations resulted in a global Clinical Dementia Rating score of 0.5 or greater. In our sample, 26.4% of participants exhibited clinical progression by this criterion 11 years post disaster—a figure notably larger than the 20.9% progression rate reported in their study after a 10-year follow-up period among older adults who were cognitively healthy at baseline. The mean (SD) age of participants in their study was 74.5 (6.7) years, indicating an older cohort compared with our sample. However, our disaster-affected cohort exhibited a higher progression rate, suggesting that factors related to disaster exposures may contribute significantly to the observed cognitive decline, beyond the effects of age alone.

Furthermore, unlike the typical cognitive trajectories observed in community-dwelling older adults, our study found a subgroup of individuals who initially exhibited low levels of cognitive disability and experienced a more rapid decline than those with higher levels of cognitive disability at baseline. This delayed-onset pattern is rarely observed in community samples, where individuals generally follow a trajectory of cognitive decline or remain stable,^25,26^ highlighting the potential for extraneous factors, such as disaster-related exposures, to accelerate cognitive decline among survivors.

Our results showed that different risk factors were associated with different cognitive trajectories, which might inform tailored intervention strategies. For instance, individuals who experienced housing damage and disruption in health care services were more likely to experience high and gradual deterioration. These findings highlight the urgent need for shelter support and timely access to health care services for those already exhibiting high levels of cognitive disability. Additionally, worsening financial conditions were linked to both the high and gradual deterioration and the low and progressive deterioration patterns, suggesting that early interventions aimed at providing financial support may be crucial in preserving the cognitive function and preventing rapid cognitive decline among older adults who survive disasters.

Ishiki et al^27^ found that among older survivors who were involuntarily relocated to temporary housing, the proportion of those deemed cognitively impaired increased significantly during the first 42 months after the disaster (24 months: 32%; 32 months: 35%; and 42 months: 38%). Other studies^7,28^ have also reported positive associations between housing loss or residential displacement and adverse cognitive outcomes. Our findings align with these studies, showing that housing damage exposure was associated with the high and gradual deterioration trajectory. Interestingly, in addition to worsening financial conditions, the composite damage score that reflects the overall disaster damage was associated with the low and progressive deterioration pattern. A possible explanation is that housing damage and worsening financial conditions were linked to an elevated risk of acute symptoms, while other disaster-related factors (eg, loss of loved ones) gradually worsened mental health,^29^ which might have subsequently aggravated cognitive health, causing delayed-onset cognitive decline.

Building on the established evidence, our study investigated the possible mechanisms through which disaster damage exposures trigger cognitive decline. Previous studies have suggested the importance of the loss of social interactions post disaster, especially for older individuals in temporary housing.^30^ Such displacement deprives survivors of opportunities for social group participation, increasing the risk of major depressive episodes by 3.79 times compared with those not relocated, according to Matsuoka et al.^31^ Additionally, the financial hardships that often follow disasters can induce significant psychological and emotional stress, adversely affecting cognitive health.^32^ This aligns with our findings that financial hardships were not associated with cognitive disability trajectories after accounting for postdisaster depressive symptoms.

Our findings provide insights into the development of various intervention strategies and emphasize the importance of a comprehensive approach to assisting older adults after disasters. The correlations between disaster damage exposures and cognitive decline suggest that interventions should address not only the immediate physical needs but also the long-term psychological and cognitive health of survivors. Cognitive training programs, mental health support, and community-based initiatives could play a critical role in mitigating cognitive decline. These interventions should be designed to foster social connections, which are vital for mental resilience and cognitive stimulation, and to provide financial support to alleviate the stress associated with postdisaster economic hardships. Moreover, the existence of different cognitive disability trajectories highlights the need for targeted interventions. These should be tailored to the specific needs and circumstances of the subgroups, recognizing that one-size-fits-all solutions might be inadequate. In addition, regular mental health assessments, including screenings for depressive symptoms, should be implemented to identify those at risk and provide timely support.

### Strengths and Limitations

This study has 3 main strengths. First, it used a natural experiment setting to address the endogeneity problem when examining the associations of disaster damage exposures with different trajectories of cognitive disability among older individuals. The availability of predisaster information allowed us to control for baseline characteristics. A second strength is that trained personnel conducted in-home visits to conduct assessments of cognitive disability. The level of housing damage was also objectively assessed by building inspectors. Finally, the study used LCGA, which provided an understanding of how within-individual cognitive disability levels changed over time. Our approach provided insights beyond what could be obtained with repeated cross-sectional analyses.

There are several limitations of the present study. First, the evaluation of cognitive assessment via home visit occurs only upon request from someone needing long-term care services. Some participants with cognitive disabilities may not have chosen to request the assessment, potentially introducing selection bias. However, it is unlikely that individuals with significant cognitive impairment would have foregone the assessment, as in-home visits are a prerequisite for receiving care services under Japan’s mandatory long-term care insurance scheme. Also, it seems unlikely that the propensity to request in-home assessment at baseline (ie, before the disaster) was correlated with the severity of disaster-related traumas, given that exposure to the effects of disaster was, by definition, not predictable. Second, selection bias could be a potential concern, given the follow-up rate of 45.8%. However, our study’s follow-up rate was comparable to that of other longitudinal research on cognitive decline involving multiple cohorts of community-dwelling older adults.^14^ It is possible that those who experienced more severe damage also had more significant cognitive decline and were more likely to drop out of the study. Although the analytic sample showed better health at baseline compared with the excluded sample, any bias introduced would likely be toward the null. Moreover, sensitivity analyses that included participants with at least 1 cognitive assessment showed no substantial change from the main analyses, demonstrating the robustness of our results.

## Conclusions

This cohort study found an increase in cognitive disability levels and identified 3 distinct cognitive disability trajectories over a decade after the disaster among community-dwelling older adults. Housing damage, worsening financial conditions, disruption in health care services, and a higher composite disaster score were positively associated with patterns of cognitive deterioration. Additionally, these results highlighted the potential role of depression in the context of disaster exposure and cognitive impairment, suggesting the importance of mental health support in the aftermath of such events.

## Notes

Supplement 1. **eTable 1.** Comparison of Baseline Characteristics Between Those Who Moved and Did Not Move in Both Analytic and Excluded Samples Among Survey Respondents (n = 5048) **eTable 2.** Criteria for Levels of Cognitive Disability in the Japanese Long-Term Care Insurance System **eTable 3.** A Composite Sleep Score Under the Self-Reported Multidimensional Sleep Health (MDSH) Framework **eTable 4.** Model Fit Parameters for Latent Class Growth Analysis With 1 to 5 Groups **eTable 5.** Model Adequacy Criterion for the 3-Group Model **eTable 6.** Associations Between Postdisaster Factors and Cognitive Trajectory by LCGA **eTable 7.** Associations Between Disaster Damage Exposures and Cognitive Trajectory for Participants With at Least 1 Cognitive Assessment During 4 Follow-Ups **eFigure 1.** Timeline of the Iwanuma Study **eFigure 2.** Trajectories of Normalized Cognitive Disability Score Identified by Latent Class Growth Analysis for Participants With at Least 1 Cognitive Assessment During 4 Follow-Ups

Supplement 2.
Data Sharing Statement
