# Supplementary material for: Long-Term Trajectories of Cognitive Disability Among Older Adults Following a Major Disaster
Source: JAMA Netw Open. 2024 Dec 2;7(12):e2448277. doi: 10.1001/jamanetworkopen.2024.48277 (PMC11612899; doi:10.1001/jamanetworkopen.2024.48277)
Supplement: Supplement 1. — eTable 1. Comparison of Baseline Characteristics Between Those Who Moved and Did Not Move in Both Analytic and Excluded Samples Among Survey Respondents (n = 5048) eTable 2. Criteria for Levels of Cognitive Disability in the Japanese Long-Term Care Insurance System eTable 3. A Composite Sleep Score Under the Self-Reported Multidimensional Sleep Health (MDSH) Framework eTable 4. Model Fit Parameters for Latent Class Growth Analysis With 1 to 5 Groups eTable 5. Model Adequacy Criterion for the 3-Group Model eTable 6. Associations Between Postdisaster Factors and Cognitive Trajectory by LCGA eTable 7. Associations Between Disaster Damage Exposures and Cognitive Trajectory for Participants With at Least 1 Cognitive Assessment During 4 Follow-Ups eFigure 1. Timeline of the Iwanuma Study eFigure 2. Trajectories of Normalized Cognitive Disability Score Identified by Latent Class Growth Analysis for Participants With at Least 1 Cognitive Assessment During 4 Follow-Ups [file jamanetwopen-e2448277-s001.pdf]

Supplementary Online Content

Hu H, Li B, Hikichi H, Kawachi I, Li X. Long-Term trajectories of cognitive disability among older adults following a major disaster. *JAMA Netw Open*. 2024;7(12):e2448277. doi:10.1001/jamanetworkopen.2024.48277

eTable 1. Comparison of Baseline Characteristics Between Those Who Moved and Did Not Move in Both Analytic and Excluded Samples Among Survey Respondents (n = 5048)
eTable 2. Criteria for Levels of Cognitive Disability in the Japanese Long-Term Care Insurance System
eTable 3. A Composite Sleep Score Under the Self-Reported Multidimensional Sleep Health (MDSH) Framework
eTable 4. Model Fit Parameters for Latent Class Growth Analysis With 1 to 5 Groups
eTable 5. Model Adequacy Criterion for the 3-Group Model
eTable 6. Associations Between Postdisaster Factors and Cognitive Trajectory by LCGA
eTable 7. Associations Between Disaster Damage Exposures and Cognitive Trajectory for Participants With at Least 1 Cognitive Assessment During 4 Follow-Ups
eFigure 1. Timeline of the Iwanuma Study
eFigure 2. Trajectories of Normalized Cognitive Disability Score Identified by Latent Class Growth Analysis for Participants With at Least 1 Cognitive Assessment During 4 Follow-Ups

This supplementary material has been provided by the authors to give readers additional information about their work.

© 2024 Hu H et al. *JAMA Network Open*.

eTable 1. Comparison of Baseline Characteristics Between Those Who Moved and Did Not Move in Both Analytic and Excluded Samples Among Survey Respondents (n = 5048)[a]

	Analytic sample, No. (%)		Excluded sample, No. (%)		
	Mover (n = 4)	Non-mover (n = 1 984)	Participants who died before the last assessment (n = 1 289)	Mover who were alive through the last assessment (n = 106)	Non-mover who were alive through the last assessment (n = 1 665)
Age, y, mean (SD)	76.5 (5.1)	72.3 (5.4)	80.0 (7.4)	75.0 (7.2)	73.7 (6.3)
Gender					
Female	1 (25.0)	828 (41.7)	663 (51.4)	37 (34.9)	664 (39.9)
Male	3 (75.0)	1 156 (58.3)	626 (48.6)	69 (65.1)	1 001 (60.1)
Educational level					
9 years or less	1 (33.3)	601 (31.2)	574 (48.4)	41 (42.3)	628 (40.0)
10-12 years	2 (66.7)	905 (46.9)	415 (35.0)	41 (42.3)	633 (40.3)
13 years or more	0 (0.0)	423 (21.9)	197 (16.6)	15 (15.5)	310 (19.7)
Marriage status					
Married	2 (50.0)	1 454 (75.7)	719 (59.8)	56 (55.4)	1 069 (68.7)
Unmarried	2 (50.0)	468 (24.3)	484 (40.2)	45 (44.6)	488 (31.3)
Equivalized income, 10 000 JPY, mean (SD)	149 (91.6)	236 (139)	223 (156)	211 (152)	224 (151)
Employment status					
Working	0 (0.0)	355 (20.1)	102 (9.4)	14 (17.3)	247 (17.3)
Not working	4 (100.0)	1 415 (79.9)	987 (90.6)	67 (82.7)	1 178 (82.7)
Cognitive disability levels	-	-	0.9 (1.7)	0.7 (1.6)	0.3 (0.9)
Self-report health status					
Excellent	1 (25.0)	248 (12.8)	104 (8.2)	9 (9.0)	190 (11.6)
Good	3 (75.0)	1 411 (72.7)	691 (54.4)	58 (58.0)	1 073 (65.3)
Fair	0 (0.0)	241 (12.4)	341 (26.9)	25 (25.0)	305 (18.6)
Poor	0 (0.0)	40 (2.06)	134 (10.6)	8 (8.0)	75 (4.6)
Number of chronic diseases	0.8 (0.5)	1.2 (0.9)	1.2 (1.0)	1.4 (1.0)	1.2 (0.9)
Receipt of medical treatment	4 (100.0)	1 480 (75.9)	1 097 (86.7)	82 (82.0)	1 292 (79.8)

[a] Ten participants without baseline cognitive assessments were removed.

eTable 2. Criteria for Levels of Cognitive Disability in the Japanese Long-Term Care Insurance System

	Criteria	Examples of observable symptoms or behaviors
Independent	No cognitive impairment. Completely independent.	
I	Suffers from cognitive decline, but daily living is almost all independent in the domestic and social spheres.	
II	Manifests some symptoms/behaviors, and communication difficulties that may hinder daily activities but can be independent if someone takes care of them.	
IIa	The abovementioned conditions in II are observed while outside the domestic sphere.	Frequently gets lost on the street or makes noticeable mistakes in matters that the person was previously able to handle, such as shopping.
IIb	The abovementioned conditions in II are also observed within the domestic sphere.	Is unable to manage taking medication or staying alone at home due to an inability to answer the phone or the door.
III	Occasionally manifests communication difficulties or symptoms/behaviors that hinder daily activities, thus requiring care.	

© 2024 Hu H et al. *JAMA Network Open*.

IIIa	Manifests abovementioned conditions described in III predominantly during daytime.	Has difficulty or takes time to change clothes, take meals, defecate, urinate; puts objects into mouth; picks up and collects objects; is incontinent, makes loud and incoherent screams, carelessly handles fire, or engages in unhygienic acts or inappropriate sexual acts, etc.
IIIb	Manifests abovementioned conditions described in III predominantly at night.	
IV	Frequently manifests difficulties in communicating or symptoms/behaviors that hinder daily activities and constantly requires care.	
M	Manifest significant mental symptoms, problematic behavior(s), or serious physical illness and require specialized medical care.	Shows continued mental symptoms such as delirium delusions; aggression; complications from physical restraints; behavior such as self-mutilation and harm to others.

© 2024 Hu H et al. *JAMA Network Open*.

eTable 3. A Composite Sleep Score Under the Self-Reported Multidimensional Sleep Health (MDSH) Framework

	Question	Response	Cutoff
Duration	How many actual hours of sleep did you get in the past month?	Number of hours (minutes)	1 point for less than 5 or more than 9
Quality	PSQI Quality Item [How would you rate your sleep quality as a whole over the past month?]	Very good (0)<br>Good (1)<br>Poor (2)<br>Very poor (3)	1 point for > 1
Disturbed sleep	How often in the past month did you take medication to help you sleep (that was prescribed by a doctor or that you bought at a pharmacy or other vendor)?	Never (0)<br>Less than once a week (1)<br>1-2 times a week (2)<br>3 or more times a week (3)	1 point for > 2

Model selection process:

This trajectory model was determined to be the best fit based on clinical insight and fitness parameters (log-likelihood = -33 842.66, AIC = 67 703.32, BIC = 67 753.67) (eTable 4). While the four-group model had a slightly better BIC value than the three-group model, the former did not include groups that were substantively distinct from those in the

© 2024 Hu H et al. *JAMA Network Open*.

latter. Thus, we selected the three-group model as the optimal solution to identify cognitive disability trajectories among participants. According to this model, the average posterior probability of accurate allocation exceeded 0.95 across all three groups for individuals allocated to each cognitive disability trajectory group. Furthermore, the proportion of observations within each group exhibiting a posterior probability greater than 0.7 likewise exceeded 95%, and the entropy approached 1. Collectively, these metrics indicate a robust classification (eTable 5).

eTable 4. Model Fit Parameters for Latent Class Growth Analysis With 1 to 5 Groups[a]

Group(s)	Log likelihood	AIC	BIC
1	-37 235.77	74 477.55	74 494.33
2	-34 925.64	69 863.29	69 896.86
3	-33 842.66	67 703.32	67 753.67
4	-33 842.66	67 709.32	67 776.46
5	-33 842.66	67 715.32	67 799.24

[a] Log likelihood is a measure of model fit with higher values indicating better fit. Akaike information criterion (AIC) and Bayesian information criterion (BIC) are measures of model fit taking into account model complexity, with lower values indicating a better fitting and more parsimonious model. AIC allows for slightly more complexity than BIC.

eTable 5. Model Adequacy Criterion for the 3-Group Model

	Mean of posterior probabilities	Posterior probabilities above 0.7 (%)	Posterior probabilities above 0.8 (%)	Posterior probabilities above 0.9 (%)	Entropy
Group 1	0.9859	99.28	98.19	97.47	0.9389882
Group 2	0.9854	98.89	98.52	97.04	-
Group 3	0.9962	99.91	99.91	99.91	-

[a] Mean of posterior probabilities is the average of the individual posterior probabilities of the subjects assigned to the group. An average posterior probability value exceeding 70% for all groups indicates adequate classification. Posterior probabilities above a certain threshold indicate the proportion of observations with a posterior probability surpassing that threshold (e.g., 0.7, 0.8, 0.9); a proportion exceeding 70% denotes strong classification confidence. Entropy is a measure of the quality of the classification of a model with values close to 1 indicating adequate classification.

eTable 6. Associations Between Postdisaster Factors and Cognitive Trajectory by LCGA

	Low and progressive deterioration		High and gradual deterioration	
	Depression symptom score [AOR (95% CI)]	Composite sleep score [AOR (95% CI)]	Depression symptom score [AOR (95% CI)]	Composite sleep score [AOR (95% CI)]
Model 1	**1.21 (1.13, 1.29)**	0.99 (0.74, 1.33)	**1.05 (1.00, 1.10)**	1.03 (0.85, 1.25)
Model 2	**1.20 (1.13, 1.29)**	0.97 (0.72, 1.31)	**1.06 (1.01, 1.11)**	1.06 (0.87, 1.28)
Model 3	**1.19 (1.11, 1.27)**	0.98 (0.73, 1.32)	1.04 (0.99, 1.09)	1.04 (0.86, 1.26)
Model 4	**1.20 (1.12, 1.28)**	0.99 (0.74, 1.32)	**1.06 (1.01, 1.11)**	1.04 (0.86, 1.26)
Model 5	**1.20 (1.12, 1.28)**	0.99 (0.74, 1.33)	**1.05 (1.00, 1.10)**	1.03 (0.85, 1.25)

Model 1 included loss of loved one(s), baseline characteristics, and postdisaster factors (depressive symptom score and composite sleep score in 2013). Model 2 included housing damage, baseline characteristics, and post-disaster factors. Model 3 included worsening financial conditions, baseline characteristics, and post-disaster factors. Model 4 included disruption in healthcare services, baseline characteristics, and post-disaster factors. Model 5 included composite damage score, baseline characteristics, and post-disaster factors. AOR = adjusted odds ratio, CI = confidence interval. Bold formatting indicates statistically significant.
Low and stable constituted the reference group.

© 2024 Hu H et al. *JAMA Network Open*.

eTable 7. Associations Between Disaster Damage Exposures and Cognitive Trajectory for Participants With at Least 1 Cognitive Assessment During 4 Follow-Ups

	Low and progressive deterioration v.s. low and stable [AOR[a] (95% CI)]	*P* value	High and gradual deterioration v.s. low and stable [AOR[a] (95% CI)]	*P* value
Loss of loved one(s)	1.02 (0.83 - 1.25)	0.87	0.77 (0.56 - 1.05)	0.09
Housing damage	1.00 (0.67 - 1.49)	0.99	1.49 (0.87 - 2.53)	0.15
Worsening financial condition	1.26 (0.98 - 1.61)	0.07	**1.51 (1.05 - 2.17)**	**0.03**
Disruption in healthcare services	1.05 (0.77 - 1.44)	0.76	**1.58 (1.04 - 2.38)**	**0.03**
Composite damage score	1.06 (0.94 - 1.18)	0.34	1.11 (0.94 - 1.30)	0.21

[a.] Adjusted for age, gender, marital status, education level, employment status, and equivalized income. N = 3854.

© 2024 Hu H et al. *JAMA Network Open*.

eFigure 1. Timeline of the Iwanuma Study

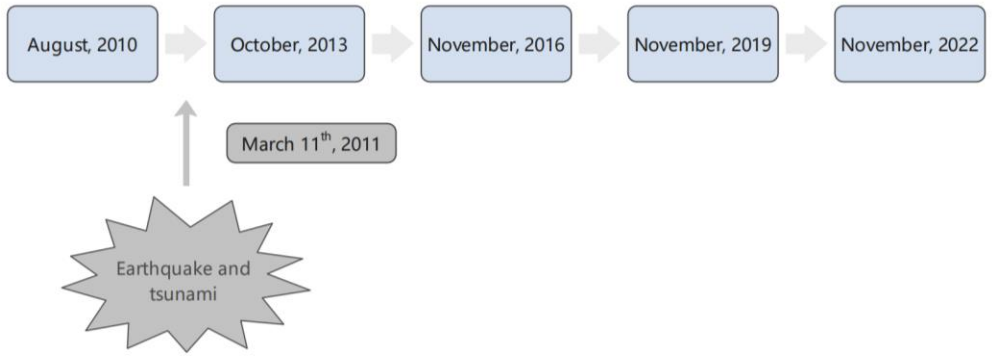

eFigure 2. Trajectories of Normalized Cognitive Disability Score Identified by Latent Class Growth Analysis for Participants With at Least 1 Cognitive Assessment During 4 Follow-Ups

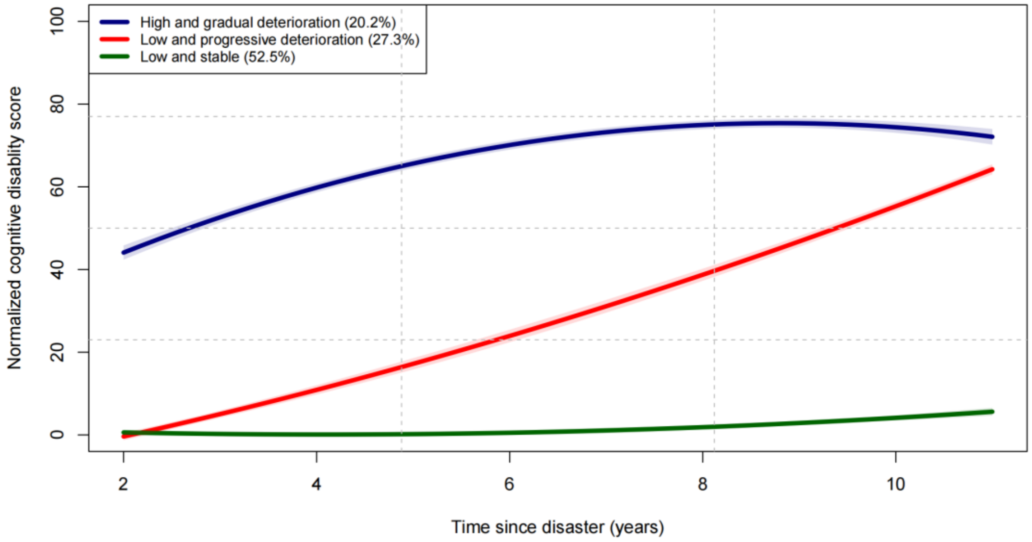

N = 3854

© 2024 Hu H et al. *JAMA Network Open*.
