# Supplementary material for: Long-Term Trajectories of Cognitive Disability Among Older Adults Following a Major Disaster
Source: JAMA Netw Open. 2024 Dec 2;7(12):e2448277. doi: 10.1001/jamanetworkopen.2024.48277 (PMC11612899; doi:10.1001/jamanetworkopen.2024.48277)
Supplement: Supplement 2. — Data Sharing Statement [file jamanetwopen-e2448277-s002.pdf]

Data Sharing Statement

Hu. Long-Term Trajectories of Cognitive Disability Among Older Adults Following a Major Disaster. *JAMA Netw Open*. Published December 02, 2024.
doi:10.1001/jamanetworkopen.2024.48277

Data
Data available: Yes
Data types: Deidentified participant data
How to access data: The data will be made available by the JAGES and Iwanuma study group.
When available: With publication

Supporting Documents
Document types: None

Additional Information
Who can access the data: Researchers whose proposed use of the data has been approved.
Types of analyses: For a specified purpose.
Mechanisms of data availability: With investigator support, after approval of a proposal, and with a signed data access agreement.
